# Closing the Implementation Gap in Precision Nursing Care

**DOI:** 10.1097/jnr.0000000000000737

**Published:** 2026-03-25

**Authors:** Hsiao-Yean CHIU

**Affiliations:** ■

The aim of precision nursing, increasingly positioned as an extension of person-centered care, is to tailor nursing interventions through the integration of biological, clinical, psychosocial, and contextual information (Ielapi et al., 2020). The successful application of precision nursing, beyond the application of related technologies, depends on the capacity of nurses to interpret biological indicators alongside patient-reported outcomes, functional status, caregiver context, and evolving care goals across the care continuum (Fu et al., 2020). However, the current evidence suggests readiness to engage in biologically informed care varies substantially among nurses, reflecting persistent gaps in data literacy, decision-support infrastructure, and role clarity, particularly in relation to genomics-informed and biomarker-informed practices (Dante et al., 2025).

Although the conceptual importance of precision nursing is well recognized, implementation in routine nursing practice remains incomplete. A major barrier is the limited availability and clinical applicability of biological and longitudinal data within nursing workflows, which constrains the ability of nurses to translate precision concepts into actionable care (Viana et al., 2021). To move precision nursing from concept to practice, clinical nursing education must be reinforced. Precision nursing competencies should be embedded systematically into undergraduate and continuing education programs through case-based learning, simulation, and practice-based coaching to enable nurses to apply biological data alongside contextual information in real-world clinical settings (Liu et al., 2023; McLaughlin et al., 2024).

Several of the articles in this issue of *The Journal of Nursing Research* illustrate both the promise and current boundaries of precision nursing. The articles examining prognostic factors for 30-day in-hospital mortality among critically ill patients receiving continuous renal replacement therapy and the influence of social support and quality of life on individuals receiving hemodialysis underscore the clinical relevance of risk stratification and tailored monitoring. The articles examining dignity in older adults enrolled in long-term care, affective touch in patients with advanced cancer, and caregiver burden and bereavement trajectories remind us that, when biological data are limited or not actionable, skilled clinical judgment and person-centered care remain central to individualized nursing practice. Collectively, these studies reinforce that advancing precision nursing across the care continuum requires not only better data but also better-prepared nurses supported by robust clinical education.
